# Relationship between fatness, physical fitness, and academic performance in normal weight and overweight schoolchild handball players in Qatar State

**DOI:** 10.1371/journal.pone.0246476

**Published:** 2021-02-19

**Authors:** Souhail Hermassi, Mohamed Souhaiel Chelly, Lars Bojsen Michalsik, Nilihan E. M. Sanal, Lawrence D. Hayes, Cristina Cadenas-Sanchez

**Affiliations:** 1 Physical Education Department, College of Education, Qatar University, Doha, Qatar; 2 Research Unit (UR17JS01) « Sport Performance, Health & Society», Higher Institute of Sport and Physical Education of Ksar-Saîd, University of “La Manouba”, Tunis, Tunisia; 3 Department of Sport Science and Clinical Biomechanics, Muscle Physiology and Biomechanics Research Unit, University of Southern Denmark, Odense, Denmark; 4 Department of Psychology, Lancaster University, Lancaster, United Kingdom; 5 School of Health and Life Sciences, University of the West of Scotland, Glasgow, United Kingdom; 6 Institute for Innovation & Sustainable Development in Food Chain (IS-FOOD), Public University of Navarra, Pamplona, Spain; Universita degli Studi di Verona, ITALY

## Abstract

The aim of this study was to examine the correlation between physiological parameters (namely fatness and physical fitness) with academic performance (namely mathematics and science grade point average [GPA]) in normal weight and overweight schoolchild handball players. Thirty-six young male team handball players (age: 9±1 years; body mass: 45.5±14.2 kg; height: 1.38±9.1 m; body fat: 19.7±5.6%) at the highest national league for their age group participated. Anthropometry was examined by measuring body mass, body fat percentage (%BF), and body mass index (BMI). Fitness testing included the Yo-Yo Intermittent Recovery Test (level 1), squat jumps (SJ) and counter-movement jumps (CMJ), and upper-limb throwing performance (2 kg medicine ball seated front throw), a 15 m sprint test, and a T-half test for change-of-direction (COD) ability. Academic performance was evaluated through school records of grade point average (GPA) of mathematics and science. BMI was negatively correlated with science GPA (r = -0.57, p<0.001) and mathematics GPA (r = -0.39, p<0.001). Significant correlations between Yo-Yo test performance and science GPA (r = 0.73, p<0.001) and mathematics GPA (r = 0.66, p<0.001) existed. T-half test score (less time taken meant a superior performance) was negatively correlated with science GPA (r = 0.48, p = 0.003) and mathematics GPA (r = 0.63, p<0.01). In conclusion, fatness and physical fitness (except for the upper-muscular strength) were significantly related to academic performance in in schoolchild handball players. Based on results of this study, it seems pragmatic and appropriate to engage young schoolchild in physical activity as it associates with superior academic performance.

## Introduction

In recent decades, research has intensified concerning methods to enhance children’s learning by altering physical activity habits and improving physical fitness. Motor ability, muscular strength, and aerobic fitness are the major components of physical fitness that demonstrate the greatest potential to enhance child health [1, 2]. Additionally, an increasing number of empirical studies indicate improved academic performance is positively correlated with physical fitness in children [2–5].

Excess body fat, typically estimated quickly and easily with body mass index (BMI), is a worldwide public health concern [2]. Obesity, defined as a BMI ≥ 30, is considered one of the fastest growing health problems in the modern world (World Health Organization) and is associated with poorer academic performance [3]. Some studies have suggested obese children have lower intelligence quotient (IQ) and exhibit poorer executive function, memory, attention, and motor skills compared with normal weight peers [6]. Recently, research has intensified concerning academic performance and its relation with body mass, physical fitness, and physical activity [3]. However, evidence regarding the relationship between obesity and academic performance remains inconclusive. Some studies have observed a positive relationship between academic performance and anthropometric composition [7, 8], whereas others have found an inverse relationship [9, 10] meaning increased body weight is inversely associated with academic performance in children. These inconsistencies may pertain to the different measures of fatness (usually assessed through BMI), and their adjustment or lack thereof for different confounding variables. For example, in some studies, BMI was categorized as a dichotomous indicator of obesity [11, 12], while in others it was treated as continuous variable [8]. Whilst BMI has limitations in predicting body fatness, other studies which measure body fat directly (using skinfold calipers, Dual-energy X-ray absorptiometry (DXA), or magnetic resonance imaging (MRI)) typically suffer from low sample sizes. Therefore, given the limited, heterogeneous and inconsistent findings on this topic, further studies examining the relationship between fatness and academic achievement are required.

Physical activity and exercise improves cardiorespiratory and musculoskeletal fitness, which are associated with increased academic achievement in children and adolescents [5, 6, 8, 13]. Specifically, physical fitness (i.e., aerobic fitness, muscular strength, and speed-agility) are considered powerful, robust measures of health in children and adolescents [14]. Previous literature is aerobic fitness centric in relation to academic achievement, concluding higher aerobic fitness is associated with superior academic achievement. The role of aerobic fitness over muscular strength and speed on driving associations with academic achievement in overweight/obese children is well described [15]. However, components such as muscular strength or speed are less investigated and there have been calls for further research in this area [16]. More research is required to test associations of different components of physical fitness (i.e. muscular strength, aerobic fitness, speed) with academic performance [17] in specific populations (i.e., handball players). Using one population for this research question controls for physical activity levels to some extent, as all players within one team in a sport will likely complete the same amount of training. Previous reports suggest aerobic fitness is the strongest correlate of academic achievement, a stronger predictor than muscular strength, endurance, and flexibility [18]. Interestingly, these authors noted that students who maintained fitness components through their time in high school outperformed those who did not maintain fitness, and this difference was of a magnitude to make a difference to the quality of high school to which they were accepted.

Recent systematic reviews found the relationship between BMI and academic achievement was stronger in American and European samples than in Asian samples, including Gulf region [19]. It is intriguing to note that among studies with Asian samples, weak relationships between BMI and academic achievement were reported [20, 21]. Data concerning countries from the Gulf region is scarce, and this geographical area has unique features, which means research from other parts of the world may not be applicable to the Gulf. Therefore, the aim of this study was to examine the correlation between fatness, different components of physical fitness, and academic performance of normal weight and overweight schoolchild handball players in Qatar State. It was hypothesized *a priori* that a strong relationship between measures of physical fitness and academic achievement would be apparent.

## Materials and methods

### Participants

Thirty-six schoolchild handball players from Doha, Qatar participated (age: 9±1 years; body mass: 45.5±14.2 kg; height: 1.38±0.90 m; body fat: 19.7±5.6%), with at least 2 years of playing experience. Participants reported no musculoskeletal injuries in the 4 weeks before the study. Players trained on average 3 times per week (i.e. 4.2 ± 0.1 h/week), in solely team handball training which consisted of motor qualities and basic technique. Matches were played once per week. Training consisted of motor skills (60% of session time) and basic team handball techniques through games (40% of session time). Subjects also engaged in weekly school physical education sessions, which lasted 40 minutes and consisted mainly of ball games. Participants did not train within 24 h of a testing session, and participants and/or guardians provided informed written consent or assent prior to study commencement. The study was conducted in accordance with the Declaration of Helsinki. The present study’s protocol was fully approved by Qatar University- institutional review board before subject recruitment and data collection (QU-IRB 1163-EA/19).

### Procedures and evaluations

Testing was conducted on an indoor handball court, from 18:00 h– 20:00 h to minimise the influence of circadian rhythms, a minimum of three days after the last competative match. The testing area was temperature controlled (20.5 ± 0.5°C, relative humidity 60 ± 5%). Participants maintained habitual nutrition, with the exception that they did not drink caffeine-containing beverages on the testing day and arrived for testing a minimum of 4 h postprandial. Players abstained from exercise training for 24 h prior to testing.

The study consisted of separate testing blocks to measure general and team handball specific physical performance characteristics [21]. The rationale for the test selection was the relevance for handball and the evaluation consisted of the following assessments:

1. Sprinting performance (10 m, 15 m),
2. Jumping performance (countermovement jump; CMJ, squat jump; SJ),
3. Throwing performance (medicine ball overhead throw),
4. Aerobic performance (Yo-Yo IR1).

After a general warm-up consisting of 5 min low intensity running, 3 × 15 m progressive accelerations, and a maximal 20 m sprint, interspersed with 3 min periods of passive recovery were performed. Warm up exercises such as trunk rotation, trunk side-bends, trunk wood-chops, internal and external rotary movements of the shoulder, push-ups and 3 to 5 free-ball throws were performed prior to the throwing tests.

After a general warm-up consisting of 5 min low intensity running, 3 × 15 m progressive accelerations, and a maximal 20 m sprint, interspersed with 3 min periods of passive recovery were performed. In addition to the low intensity running, submaximal dynamic stretches and throws were performed, consistent with our previous work [23]. Testing was conducted over four days, in a fixed order. On day one, anthropometric testing preceded vertical jump tests (SJ and CMJ), on day two, sprint testing was completed, on day three, the T-half agility test and medicine ball throw was completed, and finally on the fourth day, the Yo-Yo IR1 was conducted. To determine test-retest reliability, day one to three were repeated two weeks later. The second set of scores was considered for analysis. Anthropometric assessments and the Yo-Yo IR1 test were only completed once at the initial testing period for convenience reasons related to time and players’ schedules.

#### Day 1

*Anthropometry*. Body mass and height were measured using a portable digital scale (Tanita Body Fat Analyzer; model TBF 105; Tanita Corporation of America, Inc, Arlington Heights, Illinois). Precision of mass and height measurement were to the nearest 0.1 kg and 0.1 cm, respectively. BMI was calculated as body mass divided by the square of height (kg·m^2^). Total body fat was calculated by skinfolds assessed using a standard Harpenden caliper (Baty International, Burgess Hill, Sussex, United Kingdom). Duplicate readings were taken at each site, to the nearest 0.1 mm. If the two readings were >2 mm different, a third reading was taken, and the nearest two readings averaged. Body fat percentage was estimated using the four-site method, and sex- and age-specific equations [22], which has previously been reported in young athletes [23, 24]. The four sites used were biceps, triceps, subscapular, and suprailiac skinfolds, using the below equation:

%Bodyfat=(4.95/(Density−4.5))⋅100

WhereDensity=1.162−0.063(LOGsumof4skinfolds).

*Lower-limb muscular strength*: *Squat jump and counter movement jump tests*. During the SJ and CMJ, jump height, maximal velocity pre-take off, maximal force pre-take off, and mean power were obtained using the OptoJump photoelectronic system (Optojump Next, Microgate, Italy), with a frequency of 1 kHz, as described by Glatthorn et al [25]. Jump height was determined using contact time and flight time. Participants performed the SJ starting with knee angles at 90° without a countermovement. Participants then pushed upwards with their legs, arms akimbo, vertically. The CMJ commenced from an upright position, with participants making a rapid countermovement to a knee angle of ~90°, then pushing vertically upwards with their legs, with arms akimbo, as high as possible, as previously described [26]. The SJ and CMJ were both performed without an arm swing, and hands were fixed at the level of the pelvis. Participants were permitted four trials, interspersed by 30 s recovery, and the best performance of the four trials was recorded for analysis.

#### Day 2

*Speed*: *Sprint test*. Following warm-up, participants completed three sprints of 15 m, with a recovery period of 6–8 min. Photocells were placed at the start line, 10 m, and 15 m (Kit Racetime 2 SF, Microgate, Bolzano, Italy), 1 m above the ground. Participants started with their front foot 20 cm behind the initial photocell, and the fastest of the three trials was recorded for analysis.

#### Day 3

*Change-of-direction*: *T-half test*. Following warm-up, participants completed three trials, with a recovery period of 3 min. The T-half test was performed as previously described [27], but with a distance of 20 m, rather than 36.6 m [27], and times were recorded using photocells (Kit Racetime 2 SF, Microgate, Bolzano, Italy) 1 m above the ground. Trials commenced with participants having both feet behind the start line. Participants sprinted forward to a cone and touched it with their right hand. Facing forwards still, participants shuffled (without crossing feet) left to a second cone touching it with their left hand. Participants then shuffled right, touching a third cone with their right hand, before shuffling left to the second cone, again touching it with their left hand. Then participants sprinted backwards to the starting line, where they broke the beam of the photocells for the second time, finishing the trial. Trials were repeated if feet were crossed, participants failed to touch the base of a cone, and/or failed to face forward throughout.

*Upper-muscular strength*: *Medicine ball overhead throw*. Participants held a 2 kg medicine ball in both hands in front of the body with arms relaxed. They were instructed to throw the ball over their heads as far as possible. A countermovement was allowed during the action. Five trials were performed with a 1 min rest between trials. An average of the best four throws was subsequently used for analysis. The distance of the throw was recorded to the nearest 1 cm.

#### Day 4

*Aerobic high-intensity performance*: *The Yo-Yo Intermittent Recovery Test*, *Level 1*. The Yo-Yo IR1 was performed as described by Krustrup et al. [28]. A standardized warm-up, which comprised 5 min low-intensity activities and included jogging, lateral displacements, dynamic stretching, preceded the test. The test consisted of 20 m runs performed at increasing velocities until exhaustion, with 10 s of active recovery (2×5 m of jogging) between runs. The test consisted of 4 running bouts at 10–13 km·h^-1^ (0–160 m), then 7 runs at 13.5–14 km h^-1^ (160–440 m), then 0.5 km·h^-1^ speed increments after every 8 running bouts (i.e., after 760, 1080, 1400, 1720 m, etc.) until exhaustion. The test was terminated for a participant if they twice failed to complete the 20 m in time or felt unable to continue. The distance covered was considered as the test value. Reliability of the Yo-Yo IR1 test has been established previously [28] with a coefficient of variation (CV) of 3.7% with an intra-class correlation coefficient (ICC) of 0.92.

*Academic performance*. Academic performance was assessed through school records. To evaluate participants´ academic performance, actual Grade Point Average (GPA) along with their score (range from 0 to 100) in Mathematics and Science were obtained from the first semester of the academic year 2019–2020. The reason for only including two academic subjects was due to our interest in courses of science-related subjects. It has been reported that the academic achievement depends on academic subject, with fitness being particularly beneficial for subjects having stronger reliance on executive cognition, such as mathematics and science-related subjects [29].

### Statistical analysis

Descriptive statistics [mean, standard deviation (SD), minimum, maximum (range), and 95% confidence intervals (95% CI)] were calculated for all parameters. Pearson`s product moment correlations determined relationships between fatness (%BF, BMI), physical fitness (e.g., Yo-Yo IR1, Change-of-direction T-half test, SJ, CMJ, 10 m and 15 m sprint) and academic performance (mathematics and science), with age as a covariate. A Bonferroni correction was used to control for alpha inflation associated with multiple comparisons, whereby the alpha level was divided by the number of comparisons. Criteria adopted for interpreting the magnitude of correlations (r) between measures were: <0.1 as trivial; 0.1–0.3 as small; 0.3–05 as moderate; 0.5–0.7 as large; 0.7–0.9 as very large; and 0.9–1.0 as almost perfect as described by Portney and Watkins [29]. An *a priori* power calculation (nQuery Advisor 4.0; Statistical Solutions, Saugus, MA) was conducted with CMJ height as the primary outcome variable. Using a desired statistical power of 0.80, and an alpha level of 0.05, a two-sided test would be able to detect a change of 3.0 cm with a pooled SD of 2.0 cm [29], with a sample size of 7 per group. ICC and CV were calculated to describe intra-rater reliability of tests. Interpretation of reliability values were based on guidelines from Portney and Watkins [29], Shrout and Fleiss [30] and Hopkins [31]. An ICC above 0.75 is indicative of excellent relative reliability, and an ICC between 0.40 and 0.75 is indicative of fair-to good reliability [32]. An ICC less than 0.40 is indicative of poor reliability [28]. For intra-observer reliability, the mean intra-class correlation was reported. ICC values may be influenced by inter-subject variability of scores, because a large ICC may be reported despite poor trial-to-trial consistency if the inter-subject variability is high [30, 31]. The CV, an indicator of measurement variability and random error, was derived from log-transformed data [33]. The CV is a reliability measure with below 10% commonly used as a criterion to characterize good reliability. The 95% CIs were calculated for each CV and ICC. Statistical processing was performed using the “Statistical Package for Social Sciences” (SPSS ver. 25.0, year 2020 IBM, Armonk, NY, USA).

## Results

### Descriptive characteristics

All ICC and CV values for field tests are presented in Table 1. Table 1 also summarizes academic performance and physical fitness.

**Table 1:** Physical performance, academic achievement scores, Inter-class Correlation Coefficient (ICC) (ICC, 95% confidence limits) and Coefficient of Variation (CV) of the experimental group (n = 36).

	Mean ± SD	ICC	95%CI	CV (%)
**Anthropometry**				
BMI (kg/m^2^)	21.6 ± 4.5			
Body fat (%)	19.7 ± 5.6			
**Physical fitness**				
Aerobic performance:				
Yo-Yo test (m)	805 ± 286			
Muscular strength:				
Squat Jump (cm)	12.9 ± 2.7	0.609	0.056; 0.752	21
Counter Movement Jump (cm)	16.3 ± 3.9	0.578	0.264; 0.668	24
Medicine Ball throwing (m)	3.96 ± 0.81	0.665	0.262; 0.806	20
Speed:				
10 m (s)	3.03 ± 0.41	0.774	0.383; 0.838	14
15 m (s)	3.97 ± 0.52	0.569	-0.190; 0.688	13
Change-of-direction				
T-half test (s)	8.95 ± 1.50	0.988	0.976; 0.994	17
**Academic performance**				
Science	87.2 ± 12.4			
Mathematics	85 ± 16.7			

SD: Standard deviation; ICC: Intraclass correlation coefficient; CI: Confidence interval; CV: coefficient of variation.

### Fatness and academic performance

**Fig 1:**
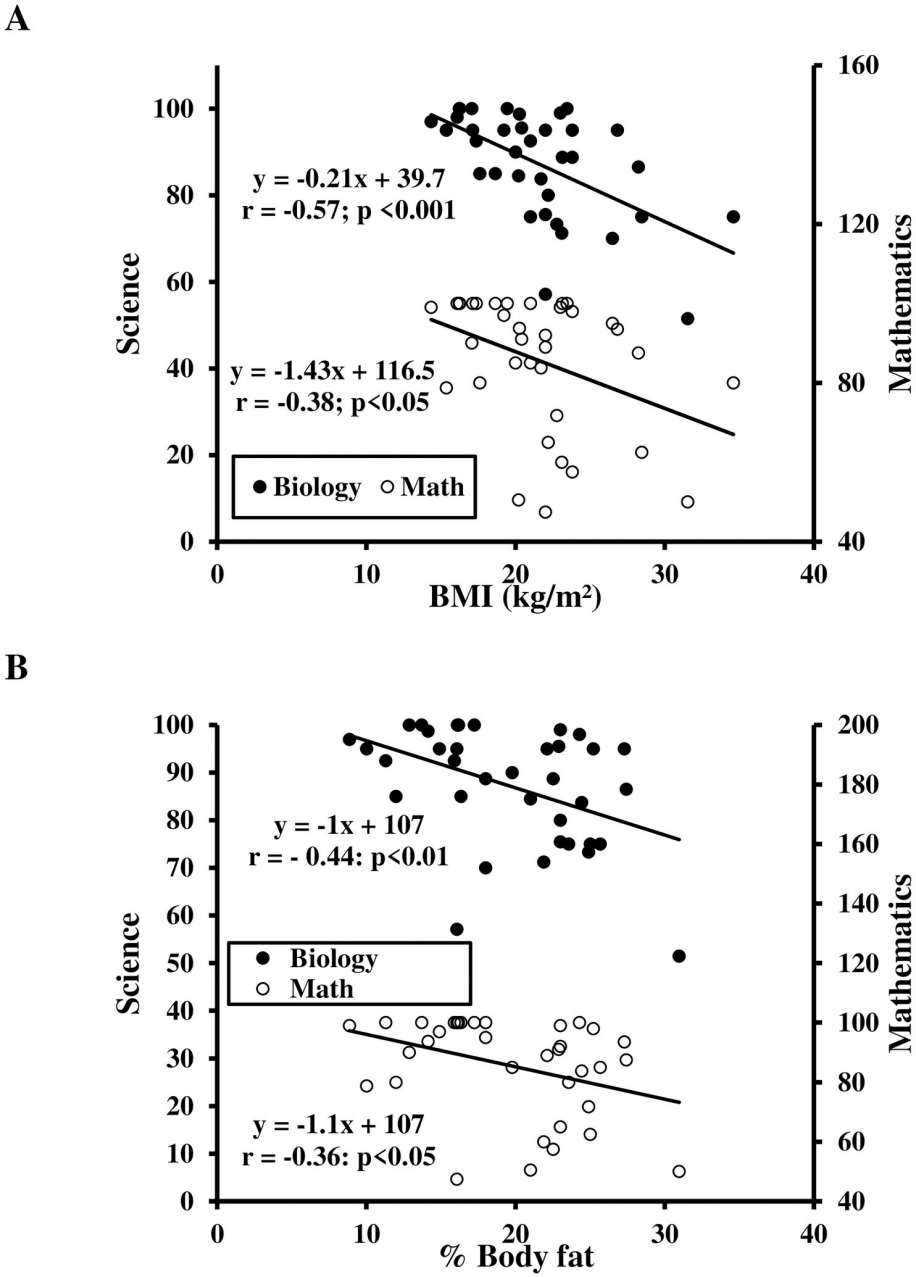
Relationship between body mass index (BMI) (A) and body fat (BF) (B) with science and mathematics scores.

Partial correlation of overweight/obesity or fatness and academic performance are presented in Table 2. BMI was negatively correlated with science GPA and mathematics GPA (Fig 1, panel A). Fig 1B depicts that %BF was significantly negatively correlated with science GPA and mathematics GPA.

**Table 2:** Correlation matrix for measure of academic and physical performances.

**BMI**	**BMI**									
**%BF**	0.713***	**%BF**								
**Yo Yo test**	-0.522***	-0.427**	**Yo Yo test**							
**SJ**	-0.435**	-0.462**	0.382	**SJ**						
**CMJ**	-0.489**	-0.461**	0.380*	0.663***	**CMJ**					
**MB**	0.101	-0.189	0.018	0.306	0.251	**MB**				
**10m**	0.119	0.126	-0.131	-0.022	-0.004	-0.093	**10m**			
**15m**	0.094	0.117	-0.028	-0.048	-0.033	-0.106	0.358*	**15 m**		
**T-Half test**	-0.370	-0.220	0.481**	0.131	0.246	-0.456**	0.066	-0.013	**T-Half test**	
**Science**	-0.573***	-0.438**	0.737***	0.518***	0.640***	0.056	-0.063	0.092	0.482**	**Science**
**Mathematics**	-0.387*	-0.359*	0.666***	0.407*	0.414*	-0.030	-0.275	-0.149	0.634***	0.720***

*: p≤0.05

**: p≤0.01

***: p≤0.001

### Physical fitness and academic performance

**Fig 2:**
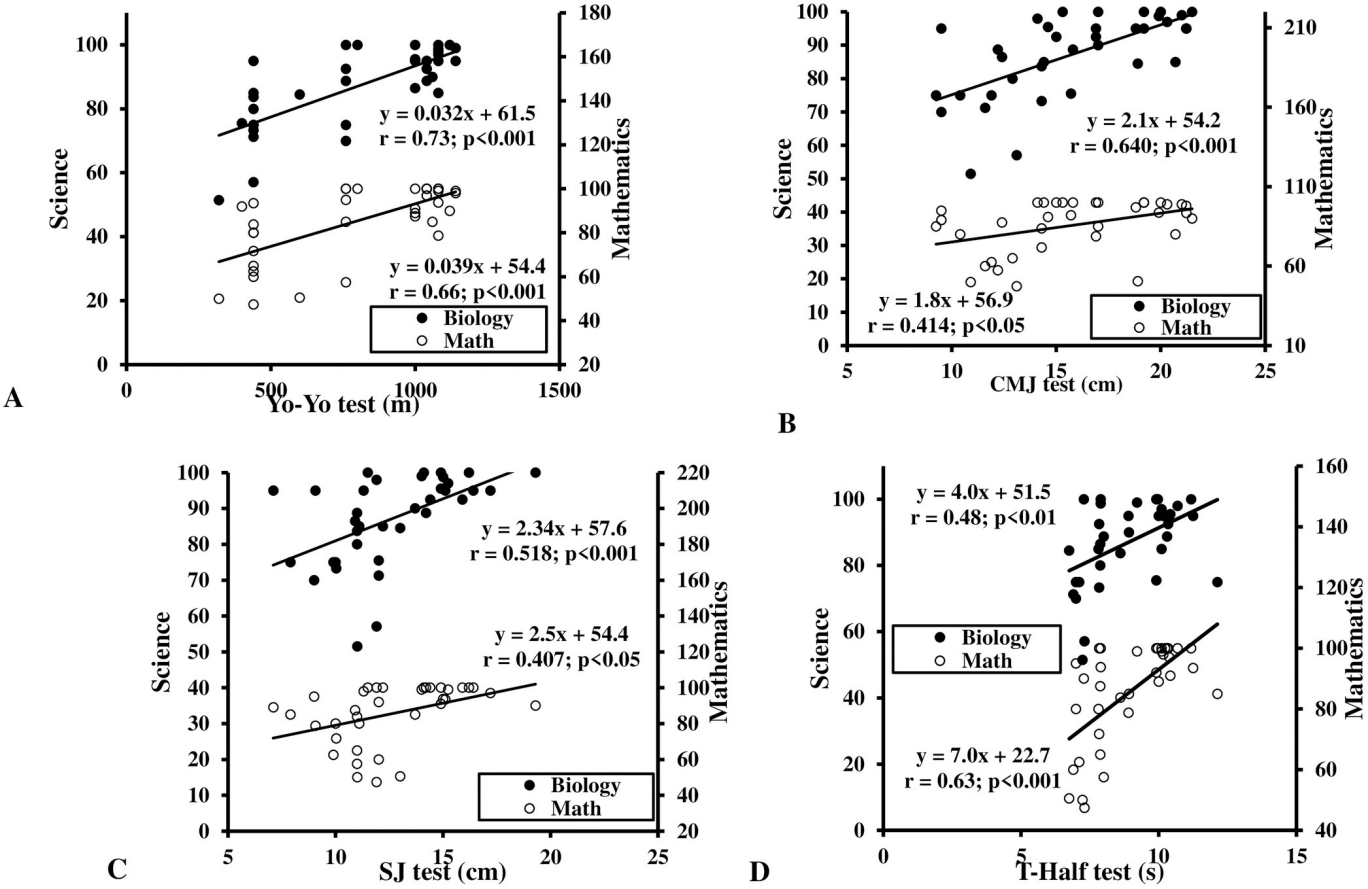
Relationship between academic scores (Science and mathematics with physical fitness variables performances: A: Aerobic score; B: Countermovement jump performance; C: Squat Jump performance; D: T-half test; SJ: Squat Jump; CMJ: Countermovement jump.

Partial correlation of physical fitness and academic performance using Pearson’s product-moment are presented in Table 2. The Yo-Yo IR1 (all r ≥ 0.67, all p < 0.001) and jump tests (all r ≥ 0.41, all p ≤ 0.05) were positively correlated with science and mathematics. Medicine ball throw and sprint values were not correlated with academic performance (all r ≤ 0.28, all p ≥ 0.103). Change-of-direction assessed by T-half test showed a positive relationship with science and mathematics (all r ≥ 0.48, all p ≤ 0.01) whilst aerobic fitness was significantly positively correlated with academic performance (all r ≥ 0.66, all p < 0.001; Fig 2A). Fig 2B and 2C shows that CMJ and SJ performance were significantly related to academic performance (all r ≥ 0.41, all p ≤ 0.014). T-half test performance was significantly positively correlated with academic performance (all r ≥ 0.48, all p ≤ 0.003; Fig 2D).

## Discussion

The main findings of this study were that correlations exist between fatness, physical fitness, and academic performance in pre-pubertal schoolchildren. Results demonstrate that both BMI and %BF were negatively correlated with academic performance. Interestingly, mathematics and science performance were more correlated to BMI than %BF. Furthermore, it was notable that aerobic fitness, lower-limb muscular strength, and change-of-direction ability had a positive, linear relationship with academic scores. In addition, lower limb muscular power measured by CMJ and SJ was positively related to academic performance. However, no correlation existed between upper-limb muscular strength or speed and academic performance.

### Relationship between fatness and academic performance

Negative associations between BMI and %BF with academic performance were found in the present study. Our work supports the supposition that children with higher fatness exhibited poorer academic performance [34, 35]. In contrast, an association between BMI and academic performance is not ubiquitous in previous investigations [7–9] as some studies reported no relationship [36, 37]. A reason for this discrepancy could be a similarity in academic performance between lowest and highest quartile BMI for sex or academic outcome measure, which implies no linear association between these variables after covariate adjustment. The present study differs from the results of Castelli et al. [38], which demonstrated lower BMI was associated with greater academic achievement. The correlation between BMI and academic performance shed light on fatness as an indicator of poor brain health (i.e. academic achievement). BMI is commonly used to define being overweight/obese but is not a specific measure of fatness.

Weight-related discrimination, commonly experienced by children with obesity, may influence self-esteem by internalizing and externalizing behavioral problems, which likely affect children’s academic performance [9, 39]. It is still unclear whether being overweight itself affects academic performance, or if this effect is mediated by other factors observed in individuals with obesity, such as poor self-esteems [40], anxiety and/or depression [41], teasing and social rejection [42], poor school attendance [43], and low physical fitness [44]. Moreover, these studies mostly use BMI as an adiposity surrogate, and it may be pertinent to examine the relationship between adipose mass specifically and academic performance.

### Relationship between physical fitness and academic performance

The present study supports previous findings from cross-sectional investigations which demonstrated fitter students had greater academic achievement [3, 4, 6]. There are several psychosocial factors which have been proposed to explicate the role of fitness in academic achievement [45]. Firstly, fitness is generally associated with superior overall health, which associates with academic achievement. Secondly, increased PA leads to improved attention and classroom behavior. Finally, superior fitness exerts positive effects on mental health and self-esteem, is associated with lower stress, anxiety, and depression, which would all positively influence academic achievement. Regarding aerobic performance, the present study results support several previous study findings. For example, Van Dusen et al. [46] found aerobic performance to be the strongest fitness component related to academic achievement, confirming previous conclusions [37, 47]. Further studies have proposed aerobic performance as a predictor of academic achievement [2, 8, 10]. These findings are manifest among children and adolescents from elementary school up to secondary school [37, 48], adding credence to the association at several ages. Mechanistically, exercise and physical activity can improve or maintain aerobic fitness which directly affects brain plasticity [49], and are associated with cognitive health, better cognitive abilities, larger brain structures [50], elevated brain function [51], improved memory [52], and superior neurocognitive function and cognitive control [20]. Improving neurocognitive functions and brain plasticity may result in better academic achievement, which has been demonstrated by Moore et al. [53]. These results emphasize the important impact of physical fitness (particularly aerobic fitness) on cognitive ability of children and adolescents.

Regarding other fitness components examined, a positive correlation between lower-limb muscular power and academic performance was demonstrated in the present study. Few studies have examined the relationship between muscular strength and academic performance, with inconclusive results. Some studies report an association between muscular strength and academic performance [46, 54]. In fact, Cadenas-Sanchez et al. [15] found a correlation between lower-limb muscular strength (i.e., 1-RM leg press) and mathematics skills measured by the Woodcock-Muñoz Muñoz test battery. This positive relationship is concordant with evidence from previous cross-sectional findings in children [47]. In contrast, other studies have reported no relationship between muscular strength and academic performance [16, 36, 48, 54, 55]. Conflicting results may be due to study sample characteristics, different strength testing methods, and different academic evaluation systems.

There is a paucity of research concerning the correlation with speed or change-of-direction ability and academic performance. In the present study, no correlation between sprint speed and academic performance was found. Yet, the positive association between change-of-direction performance and academic performance confirms data of Van Dusen et al. [46]. However, mechanisms which explain this phenomenon remain unclear. Thus, more research is needed to focus on the relationship between agility tests and academic achievement.

### Limitations of the study

There are some limitations of the present study, which we accept. The first is its cross-sectional design, which does not allow inference of temporality or causality. Secondly, the measure of fatness was only a predicted value determined via sum of skinfolds. The lack of sociodemographic and socioeconomic variables is another limitation to acknowledge. Moreover, although the sample size was modest, characteristic of the sample (i.e., team handball players) provide a novel view of the associations studied, controlling for physical activity levels to some extent. As all participants were handball players, and competed at similar levels, trained similar amounts, and played a similar number of matches, they all completed a similar level of physical activity per week, and the associations reported herein, are not merely artefacts of physical activity influencing outcome variables. Finally, other tests to determine muscular strength may be beneficial, such as a handgrip test, or an assessment of motor ability. Moreover, future investigations should consider sexual maturation when examining fatness and fitness in youths, as this may have a bearing on conclusions drawn.

## Conclusion

The present findings support the assumption that correlations exist between fatness, fitness, and academic performance in pre-pubertal handball schoolchildren. Based on associations presented here, it seems pragmatic to promote physical fitness in this age group. This would likely be done via exercise or physical activity, which in turn could reduce excess adiposity, increase physical fitness, and improve academic performance. However, future intervention studies with large sample sizes are needed to corroborate our findings, and establish temporality and causality.

## Supporting information

S1 Data (XLSX)
